# Distinct patterns of speech disorder in early-onset and late-onset de-novo Parkinson’s disease

**DOI:** 10.1038/s41531-021-00243-1

**Published:** 2021-11-11

**Authors:** Jan Rusz, Tereza Tykalová, Michal Novotný, Evžen Růžička, Petr Dušek

**Affiliations:** 1 Department of Circuit Theory, Faculty of Electrical Engineering, Czech Technical University in Prague, Prague, Czech Republic; 2 Department of Neurology and Centre of Clinical Neuroscience, First Faculty of Medicine, Charles University, Prague, Czech Republic

**Keywords:** Parkinson's disease, Neurological manifestations, Parkinson's disease

## Abstract

Substantial variability and severity of dysarthric patterns across Parkinson’s disease (PD) patients may reflect distinct phenotypic differences. We aimed to compare patterns of speech disorder in early-onset PD (EOPD) and late-onset PD (LOPD) in drug-naive patients at early stages of disease. Speech samples were acquired from a total of 96 participants, including two subgroups of 24 de-novo PD patients and two subgroups of 24 age- and sex-matched young and old healthy controls. The EOPD group included patients with age at onset below 51 (mean 42.6, standard deviation 6.1) years and LOPD group patients with age at onset above 69 (mean 73.9, standard deviation 3.0) years. Quantitative acoustic vocal assessment of 10 unique speech dimensions related to respiration, phonation, articulation, prosody, and speech timing was performed. Despite similar perceptual dysarthria severity in both PD subgroups, EOPD showed weaker inspirations (*p* = 0.03), while LOPD was characterized by decreased voice quality (*p* = 0.02) and imprecise consonant articulation (*p* = 0.03). In addition, age-independent occurrence of monopitch (*p* < 0.001), monoloudness (*p* = 0.008), and articulatory decay (*p* = 0.04) was observed in both PD subgroups. The worsening of consonant articulation was correlated with the severity of axial gait symptoms (*r* = 0.38, *p* = 0.008). Speech abnormalities in EOPD and LOPD share common features but also show phenotype-specific characteristics, likely reflecting the influence of aging on the process of neurodegeneration. The distinct pattern of imprecise consonant articulation can be interpreted as an axial motor symptom of PD.

## Introduction

Parkinson’s disease (PD) is a neurodegenerative disorder with pathological deposits of α-synuclein, leading to the loss of dopaminergic neurons in the substantia nigra, which is the direct cause of principal motor manifestations including bradykinesia, rigidity, and resting tremor^1^. The risk of developing PD is gender and age-dependent, with incidence rate reported to be approximately 3.4 per 100,000 person-years in those aged under 50 years, compared to the overall incidence rates 37.6 and 61.2 per 100,000 person-years in females and males respectively^2^. According to the age at beginning of symptoms, PD patients can be subdivided into early-onset PD (EOPD) and late-onset PD (LOPD). Although consistent definition is lacking, broadly defined EOPD includes onset at or before the age of 50 years and LOPD undoubtedly includes onset at or above the age of 70 years^2–5^. LOPD tends to progress more rapidly, with patients typically presenting greater severity of non-motor and axial symptoms including gait disorder than EOPD patients^6–8^. On the other hand, EOPD comprises greater proportion of genetic forms, and is associated with a higher incidence of motor complications^2,3^. All these aspects highlight phenotypic differences between EOPD and LOPD^8^.

Hypokinetic dysarthria, developing in up to 90% of PD patients during the course of the disease^9^, is a complex motor speech impairment characterized mainly by dysphonia, imprecise articulation, and dysprosody (monopitch, monoloudness, and various timing abnormalities) with respiration problems also may present^10^. PD patients manifest substantial variability in severity of dysarthric patterns across these speech subsystems^11,12^. Specific speech abnormalities may be, thus, associated with distinct clinical phenotypes. In particular, a recent study showed that speech impairment was more pronounced in the postural instability/gait difficulty (PIGD) motor subtype compared to the tremor dominant subtype^13^. In addition, several previous studies have reported a relationship between speech and gait disorders^14–16^. Considering that LOPD presents with more expressed gait disturbances^17^, we may hypothesize that certain speech abnormalities will be detectable only in LOPD. However, nothing is known about the prevalence and patterns of speech abnormalities in EOPD compared to LOPD. Yet, the knowledge about possible speech differences between EOPD and LOPD could be used to develop a more efficient personalized approach for speech therapy strategies in PD individuals.

The current study aimed to compare speech disorder in patients with de-novo, drug-naïve EOPD and LOPD relative to age- and sex-matched young and old healthy control groups to test the hypothesis of whether PD subgroups would manifest different patterns of dysarthria.

## Results

### Clinical characteristics

The EOPD group consisted of 24 patients (15 men) with a mean age of 45.1 (SD 5.4, range 34–52) years and the LOPD group included 24 patients (14 men) with a mean age of 75.4 (SD 3.1, range 71–81) years. The YHC group consisted of 24 participants (15 men) with a mean age of 45.2 (SD 5.6, range 35–52) years, whereas OHC group included 24 participants (14 men) with a mean age of 75.5 (SD 3.2, range 71–81) years. Compared to patients with EOPD, patients with LOPD had significantly shorter symptom duration, higher PIGD score, higher number of comorbidities and overall higher severity on non-motor symptoms including higher prevalence of rapid eye movement sleep behavior disorder, lower MoCA score, and higher SCOPA-AUT score (Table 1).

**Table 1:** Demographic and clinical characteristics for PD groups.

	EOPD (*n* = 24)	LOPD (*n* = 24)	*p*-value
*Demographics*			
Male sex	15 (63%)	14 (58%)	0.77
Age (years)	45.1 (SD 5.4, range 34–52)	75.4 (SD 3.1, range 71–81)	<0.001
Age at onset (years)	42.6 (SD 6.1, range 30–50)	73.9 (SD 3.0, range 70–81)	<0.001
Symptom duration (years)	2.6 (SD 1.6, range 0.9–6.2)	1.5 (SD 1.2, range 0.3–5.9)	0.007
Positive family history of PD	5 (21%)	1 (4%)	0.08
*Motor symptoms*			
MDS-UPDRS part III	27.0 (SD 11.7, range 6–56)	32.7 (SD 8.5, range 16–52)	0.06
Bradykinesia score	15.1 (SD 7.3, range 2–35)	17.7 (SD 6.3, range, 6–34)	0.14
Rigidity score	4.1 (SD 2.9, range 1–11)	3.4 (SD 1.9, range 0–7)	0.67
Tremor score	5.0 (SD 2.7, range 1–9)	6.7 (SD 3.5, range 1–15)	0.11
PIGD score	1.2 (SD 1.2, range 0–5)	2.6 (SD 1.4, range 1–6)	<0.001
Speech score	0.42 (SD 0.50, range 0–1)	0.63 (SD 0.49, range 0–1)	0.16
*Non-motor symptoms*			
RBD presence	1 (4%)	8 (33%)	0.01
MoCA score	26.3 (SD 2.7, range 20–30)	23.5 (SD 2.4, range 19–28)	<0.001
SCOPA-AUT score	5.8 (SD 3.4, range 0–11)	10.5 (SD 4.4, range 2–20)	<0.001
*Comorbidities*			
Vascular risk comorbidities score #	0.46 (SD 0.72, range 0–2)	1.42 (SD 1.06, range 0–4)	0.01

Data are mean (standard deviation, range) including *p*-values analyzed using Mann–Whitney U test or number (%) including *p*-values analyzed using Chi-square test. # Include presence of history of arterial hypertension, diabetes, atrial fibrillation, prior ischemic stroke, hypercholesterolemia, and current smoking status.

*EOPD* = early-onset Parkinson’s disease, *LOPD* = late-onset Parkinson’s disease, *PD* = Parkinson’s disease; *MDS-UPDRS* = Movement Disorder Society Unified Parkinson’s Disease Rating Scale, *PIGD* = postural instability/gait difficulty, *RBD* = rapid eye movement sleep behavior disorder, *MoCA* = Montreal Cognitive Assessment, *SCOPA-AUT* = Scales for Outcomes in Parkinson’s Disease - Autonomic Dysfunction.

### Phenotype-specific speech characteristics

**Fig. 1:**
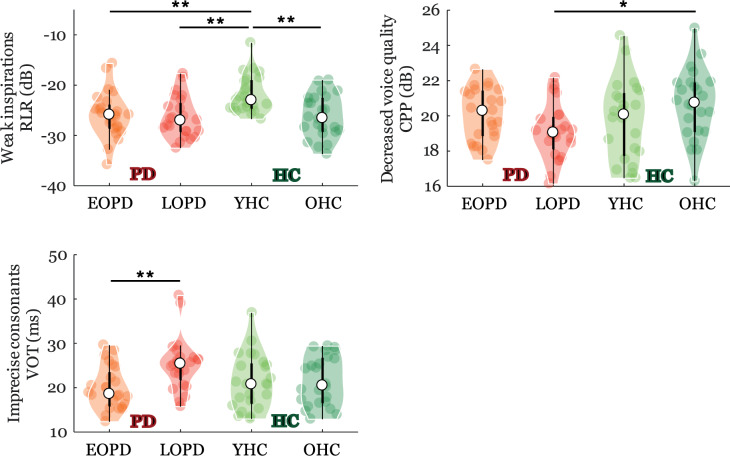
Violin plots of phenotype-specific speech characteristics. The plot shows the median (indicated by the black open circle), the interquartile range (the thick, solid vertical band), estimator of the density (color vertical curves) of the individual scores in each group (comparable to a box plot, except that the distribution of values is illustrated as density curves), and individual scores (color filled circles). Statistically significant differences between groups: *p < 0.05; **p < 0.01, ***p < 0.001. *HC* = healthy controls, *PD* = Parkinson’s disease; *EOPD* = early-onset Parkinson’s disease; *LOPD* = late-onset Parkinson’s disease; *YHC* = young healthy controls; *OHC* = old healthy controls; *RLR* = relative loudness of respirations; *CPP* = cepstral peak prominence; *VOT* = voice onset time.

Age-related differences in PD group only were reflected by significant GROUP × AGE interactions and detected for weak inspirations (RLR: *p* = 0.03), decreased voice quality (CPP: *p* = 0.01), and imprecise consonants (VOT: *p* = 0.03) (Fig. 1). According to the post-hoc tests, weak inspirations were observed in EOPD, which showed worse performance compared to its young counterparts (i.e., YHC) but similar performance to both older groups of LOPD and OHC. Decreased voice quality and imprecise consonants were observed only in LOPD, while the remaining three groups of EOPD, YHC, and OHC manifested comparable performance.

### Parkinsonian-specific speech characteristics

**Fig. 2:**
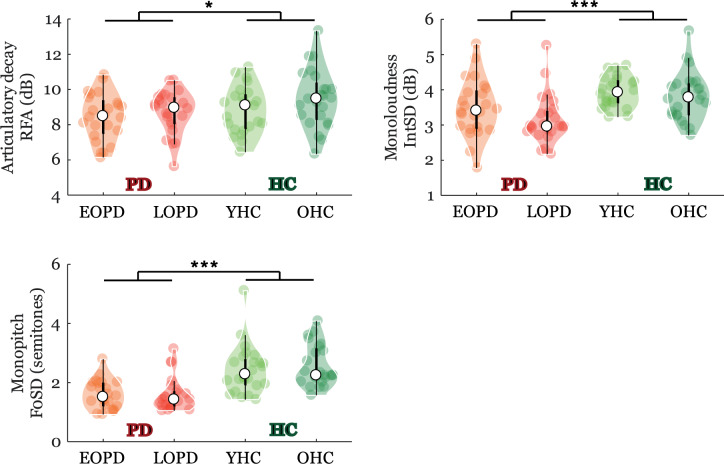
Violin plots of parkinsonian-specific speech characteristics. The plot shows the median (indicated by the black open circle), the interquartile range (the thick, solid vertical band), estimator of the density (color vertical curves) of the individual scores in each group (comparable to a box plot, except that the distribution of values is illustrated as density curves), and individual scores (color filled circles). Statistically significant differences for GROUP effect: *p < 0.05; **p < 0.01, ***p < 0.001. *HC* = healthy controls, *PD* = Parkinson’s disease; *EOPD* = early-onset Parkinson’s disease; *LOPD* = late-onset Parkinson’s disease; *YHC* = young healthy controls; *OHC* = old healthy controls; *RFA* = resonant frequency attenuation; *IntSD* = intensity variability; *F0SD* = fundamental frequency variability.

**Fig. 3:**
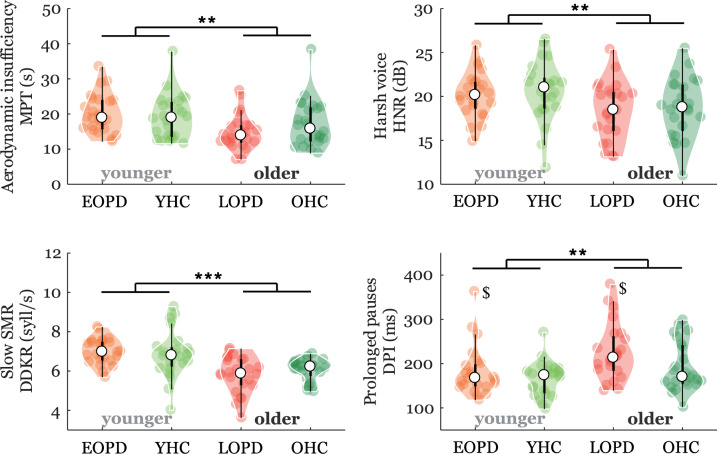
Violin plots of age-specific speech characteristics. The plot shows the median (indicated by the black open circle), the interquartile range (the thick, solid vertical band), estimator of the density (color vertical curves) of the individual scores in each group (comparable to a box plot, except that the distribution of values is illustrated as density curves), and individual scores (color filled circles). Statistically significant differences for AGE (at disease onset) effect: *p < 0.05; **p < 0.01, ***p < 0.001. ^$^Significant GROUP effect: *p* = 0.03. *HC* = healthy controls, *PD* = Parkinson’s disease; *EOPD* = early-onset Parkinson’s disease; *LOPD* = late-onset Parkinson’s disease; *YHC* = young healthy controls; *OHC* = old healthy controls; *MPT* = maximum phonation time; *HNR* = harmonics-to-noise ratio; *DDKR* = diadochokinetic rate; *DPI* = duration of pause intervals; *SMR* = sequential motion rates.

Significant GROUP differences between PD and controls were found for articulatory decay (RFA: *p* = 0.04) as well as both prosodic parameters of monoloudness (IntSD: *p* < 0.001) and monopitch (F0SD: *p* < 0.001) (Fig. 2) but also for prolonged pauses (DPI: *p* = 0.03) (Fig. 3).

### Age-specific speech characteristics

Significant AGE differences between younger and older groups of participants were found for aerodynamic insufficiency (MPT: *p* = 0.003), harsh voice (HNR: *p* = 0.006), as well as both timing parameters of slow SMR (DDKR: *p* < 0.001) and prolonged pauses (DPI: *p* = 0.002) (Fig. 3).

### Correlation between speech and motor subscores

The extent of imprecise consonant articulation (VOT) was correlated to PIGD score (*r* = 0.38, *p* = 0.008) but not to bradykinesia (*r* = 0, *p* = 0.98) or rigidity (*r* = −0.11, *p* = 0.45) scores. No other significant correlations between speech and PIGD, bradykinesia or rigidity scores were detected.

## Discussion

This study strives to determine phenotypes of speech disorder based on the age of the PD onset. The strength of this study is that speech features were evaluated in untreated newly diagnosed PD patients with a simultaneous evaluation using objective and blinded (fully-automated) acoustic analysis. Examining drug-naïve patients is especially important as dopaminergic treatment may improve certain aspects of speech disorder^18^, and thus alter the natural phenotypic-based speech differences. In particular, we were able to uncover three phenotype-specific speech characteristics differing between EOPD and LOPD. Despite similar perceptual dysarthria severity in both PD subgroups, EOPD showed weaker inspirations, while LOPD was characterized by decreased voice quality and imprecise consonant articulation. Also, this study highlighted three specific characteristics of hypokinetic dysarthria including monopitch, monoloudness and articulatory decay that were consistently presented in both PD phenotypes and were not related to ageing.

The distinctive speech phenotype of PD with an onset in older age may relate to various factors. A different natural course of LOPD is likely caused by accelerated spread of neurodegenerative pathology in the elderly and by gradual decrease of nigral dopaminergic neurons naturally occurring during aging^19^. Both imprecise consonant articulation and decreased voice quality represent rather non-specific markers of neuronal dysfunction that are typically more prevalent in atypical parkinsonism with more severe brain atrophy^20,21^. Thus, the higher severity of these speech characteristics observed in LOPD compared to EOPD may also be attributed to more widespread neurodegeneration. Furthermore, brain of elderly PD patients may possess reduced capacity of compensatory mechanism leading to a more pronounced impairment with more rapid disease progression that affects speech^22^. Last but not least, prevailing evidence suggests that impairment of temporal speech dimensions in hypokinetic dysarthria are related to axial motor symptoms^23,24^. Therefore, the decreased consonant articulation examined via voicing onset time, which represents a temporal measure of coordination of speech articulation and voicing, may be partly related to generally higher severity of axial motor symptoms observed in LOPD. This assumption is further supported by the detected relationship between imprecise consonant articulation and PIGD score, which cannot be interpreted as a simple effect of increased motor severity as no relationship to bradykinesia and rigidity score was observed.

Interestingly, compared to corresponding controls, our EOPD patients showed weak inspirations that were reflected by a higher relative loudness measured between inspirations and speech (i.e., inspirations had lower loudness compared to average loudness of speech). Such a difference in the loudness of respiration was not seen between LOPD and corresponding controls. This lack of difference might be explained by a natural aging process as a decrease of maximal inspiratory pressure was observed in elderly over the age 65^25^. To the best of our knowledge, no previous study strived to assess inspiration characteristics during a natural connected speech in de-novo PD. The finding of weak inspirations in our EOPD patients is in agreement with a recent study showing that inspiratory muscle strength appears to be impaired in early-stage PD patients with an average disease duration of 1.9 years and a relatively young age of 61.7 years^26^. However, the distinction of respiratory and phonatory systems and their relative contribution in hypokinetic dysarthria in PD is still debatable. In particular, rigidity of the intercostal muscles can affect respiration, but the amount of breath needed for speech is around 10%^27–30^.

While not directly investigated before, the age-independent occurrence of monopitch, monoloudness, and articulatory decay observed in both PD subgroups is in accordance with the landmark perceptual descriptions of distinctive patterns of hypokinetic dysarthria^31,32^. As these speech dimensions are consistently impaired in PD and at the same time not affected by aging, they might provide useful biomarkers for an early diagnosis of parkinsonism. In particular, monopitch appear to be language-independent and can be detected even in patients with idiopathic rapid eye movement sleep behavior disorder^33^, which is considered the strongest marker of prodromal synucleinopathy and predictor of future conversion to PD^34^. In addition, acoustic estimation of monopitch was found to be a robust measure resistant to low microphone quality and may be thus assessed using a smartphone from patients’ home^35^. Collecting speech data through mobile devices attracts increasing attention of the community investigating PD biomarkers^36–38^, with the motivation to aid the recruitment into large studies examining innovative therapies for prodromal PD and to enable rapid access to neuroprotective therapy once available.

The remaining speech dimensions, including aerodynamic insufficiency, harsh voice, slow SMR, and prolonged pauses, appeared to reflect the natural aging process, with older participants manifesting worse performance compared to younger participants. Although most of these speech dimensions have been previously found to be affected early in the course of PD^11,33^, their impairment, at least to some degree, might be induced by aging and not just by the disease itself. Indeed, the significant effect of age on measures reflecting phonation time, voice harshness, articulation rate, and pauses has already been demonstrated in studies examining the effect of healthy aging on speech^39–41^. Overall, these findings might have implications for potential future clinical trials in which PD participants should be well-stratified according to the age should these speech dimensions represent an outcome measure.

One potential limitation is that EOPD patients had a significantly longer disease duration than the LOPD patients. This might be caused by more rapid disease progression in the LOPD group, leading to a faster development of motor symptoms and thus to earlier establishment of the diagnosis. Also, our MDS-UPSRS part III score in both EOPD (mean 27.0, SD 11.7) and LOPD (mean 32.7, SD 8.5) groups tends to be higher than reported in Parkinson’s Progression Markers Initiative Cohort (PPMI, mean 20.7, SD 8.9)^42^. Although the inclusion criteria in our BIO-PD study^43^ were very similar to that in the PPMI trial, we did not exclude patients with subjective symptom duration above 24 months. Longer symptom duration at the diagnosis could have contributed to a higher MDS-UPDRS III score, although we did not observe a significant correlation between symptom duration and MDS-UPDRS III score. Another possibility is that higher MDS-UPDRS part III scores in our study are due to a stricter rating. Importantly, MDS-UPDRS part III mean score and its dispersion in our study are comparable to values reported in the ICICLE-PD study (mean 27.6, SD 11.9)^44^. In addition, the range of our MDS-UPDRS part III score (i.e., 6–56) is also comparable to the DeNoPa study (i.e., 3–53)^45^, despite it used the older UPDRS score for motor symptoms rating. Finally, the MoCA scores in both EOPD and LOPD groups were relatively low, considering that the typical cut-off for cognitive impairment in PD patients is 26/27 points. Notably, several validation studies have challenged the universal MoCA cut-off score for cognitive impairment in PD^46^. It was shown that this cut-off value is dependent on cultural and language bias, age, and education^47^. In the normative study of the Czech MoCA version examining a cohort of 540 elderly subjects aged from 60 to 96 years, the mean MoCA score was 24.7 (SD 2.9)^47^, which is comparable to a previous study on Czech PD patients with mean MoCA score of 24.8 (SD 3.5)^48^, and also to our current study where mean MoCA score was 26.3 (SD 2.7) for EOPD and 23.5 (SD 2.4) for LOPD.

In summary, the present study demonstrates intriguing advances in the use of acoustic analysis to distinguish various PD phenotypes. While decreased voice quality and imprecise consonant articulation were specific for the LOPD, weak inspirations were only presented in the EOPD. We also suggest using monopitch, monoloudness, and articulatory decay as universal and easy to interpret markers of motor speech impairment in PD. Further exploration of the pathophysiologic differences among PD speech phenotypes defined according to the gender^23^ and/or different clinical criteria^49^ is warranted to shed light on the underlying mechanisms of dysarthria. Future longitudinal studies are needed to track pathological and clinical correlates of distinctive speech patterns across disease progression in early- and late-onset PD.

## Methods

### Participants

From 2016 to 2021, a consecutive group of de-novo, drug-naive Czech native PD patients were recruited. PD patients were diagnosed based on the Movement Disorder Society clinical diagnostic criteria for PD^50^ and investigated before the introduction of pharmacotherapy. This study is part of a longitudinal project “biomarkers in PD (BIO-PD)” aimed to collect a large representative sample of de-novo PD patients; the detailed protocol of this project has been described previously^43^. The inclusion criteria for PD were as follows: (i) age at onset below 50 or above 70 years, (ii) native Czech language speaker, (iii) no history of therapy with antiparkinsonian medication, (iv) no history of communication or significant neurological disorders unrelated to PD, and (v) no current involvement in any speech therapy. The exclusion criteria were as follows: (i) treatment with antiparkinsonian medication before baseline examination, (ii) clinical or imaging signs of atypical parkinsonism, (iii) normal finding on dopamine transporter single-photon emission computed tomography examination, and (iv) cognitive impairment that could affect the performance of speech protocol. PD patients were categorized into two groups based on their age at onset of the first motor symptom related to PD, i.e., resting tremor, bradykinesia, or rigidity. The EOPD group included patients with age at onset ≤ 50 years while LOPD group consisted of patients with age at onset ≥ 70 years. In addition, young healthy control (YHC) group age- and sex-matched to the EOPD group and old healthy control (OHC) group age- and sex-matched to the LOPD group were enrolled. The control subjects were recruited from the general community through advertisements. To be eligible for the study, controls had to be free of speech disorder, motor neurologic disorder, active oncologic illness, and abuse of psychoactive substances.

The study was approved by the Ethics Committee of the General University Hospital in Prague, Czech Republic and have therefore been performed in accordance with the ethical standards laid down in the 1964 Declaration of Helsinki and its later amendments. All participants provided written, informed consent to the neurological examination and recording procedure.

### Clinical examination

The clinical evaluation of each subject included (i) structured clinical interview focused on personal and medical history, history of drug and substance intake and current drug usage, (ii) quantitative testing of PD motor symptoms using the Movement Disorder Society-Unified Parkinson Disease Rating Scale (MDS-UPDRS) part III^51^, (iii) video-polysomnography, (iv) cognitive testing with the Montreal Cognitive Assessment (MoCA)^52^, and (v) autonomic symptoms evaluation with the Scales for Outcomes in Parkinson’s Disease-Autonomic Dysfunction scale (SCOPA-AUT)^53^. Based on MDS-UPDRS part III, composite scores including bradykinesia (sum of items 3.4–3.8 and 3.14), rigidity (sum of items 3.3), tremor (sum of items 3.15–3.18), and PIGD (sum of items 3.9–3.13) were calculated. Perceptual speech severity was estimated using speech item of the MDS-UPDRS part III (item 3.1). All diagnoses and evaluations of clinical scales were performed by a neurologist experienced in movement disorders and certified for the MDS-UPDRS usage (P.D.). Symptom duration was estimated based on the self-reported occurrence of the first motor symptoms. Based on the known vascular risk factors^54^, we also calculated the vascular risk comorbidities score, including the history of arterial hypertension, diabetes, atrial fibrillation, prior ischemic stroke, hypercholesterolemia, and current smoking status (i.e., range 0–6).

### Speech examination

Speech recordings were performed in a quiet room with a low ambient noise level using a head-mounted condenser microphone (Beyerdynamic Opus 55, Heilbronn, Germany) placed approximately 5 cm from the subject’s mouth. Speech signals were sampled at 48 kHz with 16-bit resolution. Each subject was recorded during a single session with a speech specialist. All participants were instructed to perform three vocal tasks of (i) sustained phonation of the vowel /a/ per one breath for as long and steadily as possible, (ii) fast /pa/-/ta/-/ka/ syllable repetition at least seven times per one breath, (iii) reading a short paragraph of standardized text composed of 80 words, and (iv) monologue on a self-chosen topic for approximately 90 s. These speaking tasks were chosen as they can provide comprehensive information necessary for the objective description and interpretation of motor speech disorders^10,55^. Sustained phonation, fast syllable repetition, and reading passage were performed two times per session for every subject.

### Acoustic speech analysis

We performed a quantitative acoustic vocal assessment of 10 distinct speech dimensions related to respiration, phonation, articulation, prosody, and speech timing. Acoustic analysis was preferred because it provides objective, sensitive and quantifiable information for the precise assessment of speech performance from very early stages of PD^33^. Considering respiratory dimensions, we obtained *aerodynamic insufficiency* using the maximum phonation time (MPT) via sustained phonation and *weak inspirations* using the relative loudness of respiration (RLR) via monologue. To assess phonatory dimensions, we examined *harsh voice* using the harmonics-to-noise ratio (HNR) via sustained phonation and *decreased voice quality* using the cepstral peak prominence (CPP) via monologue. To investigate articulatory characteristics, we extracted *imprecise consonants* using the voice onset time (VOT) via syllable repetition and *articulatory decay* using the resonant frequency attenuation (RFA) via monologue. With respect to prosodic characteristics, we calculated *monoloudness* using the standard deviation (SD) of intensity contour (IntSD) and monopitch using the standard deviation of pitch contour (F0SD), both via reading passage; reading was preferred as a different monologue subject chosen by each patient can influence prosodic aspects of speech. Considering timing characteristics, we computed *slow sequential motion rates* using the diadochokinetic rate (DDKR) via syllable repetition and *prolonged pauses* using the duration of pause intervals (DPI) via monologue. The final speech values used for the statistical analyses were averaged across two repetitions to provide greater speech assessment stability^55^. The definitions of these 10 acoustic parameters are summarized in Table 2. Comprehensive algorithmic details on individual acoustics measures have been reported previously^56^. Also, the accuracy of algorithms for the identification of glottal cycles, temporal intervals, and pitch sequence has been thoroughly tested in previous studies^56–58^. All analyses were performed in MATLAB® (MathWorks, Natick, MA).

**Table 2:** Overview of applied acoustic measurements.

Deviant speech dimension [vocal task]	Acoustic feature	Definition	Pathophysiological interpretation with respect to hypokinetic dysarthria
**Respiration**			
Aerodynamic insufficiency [sustained phonation]	MPT	Maximum phonation time, defined as the maximum duration of sustained vowel phonation.	The rigidity of respiratory muscles leads to decrease ability to sustain vowel.
Weak inspirations [monologue]	RLR	Relative loudness of respiration, defined as the median of loudness measured relatively between respirations and speech as a difference in logarithmic scale.	Hypokinesia of respiratory muscles and decreased range of rib cage motion make respiration quieter.
**Phonation**			
Harsh voice [sustained phonation]	HNR	Harmonics-to-noise ratio, defined as the amount of noise in the speech signal.	Reduced rate of airflow and improper control of vocal folds causes increased turbulent noise.
Decreased voice quality [monologue]	CPP	Cepstral peak prominence, defined as the measure of cepstral peak amplitude normalized for overall amplitude.	Deteriorated control of laryngeal muscles leads to unstable periods of vocal fold opening, causing a dysphonic and breathy voice.
**Articulation**			
Imprecise consonants [syllable repetition]	VOT	Voice onset time, defined as the length of the entire consonant from initial burst to vowel onset.	Hypokinesia causes slowing of lip and tongue movements, leading to a longer time required to pronounce individual consonants.
Articulatory decay [monologue]	RFA	Resonant frequency attenuation, defined as the differences between the maxima of the second formant region and minima of local valley region called antiformant.	Hypokinesia leads to decrease spectral energy as a result of decayed articulatory movements.
**Prosody**			
Monoloudness [reading passage]	IntSD	The standard deviation of speech intensity contour extracted from voiced segments.	Hypokinesia leads to the decreased amplitude of respiratory and thyroarytenoid muscles.
Monopitch [reading passage]	F0SD	The standard deviation of fundamental frequency contour converted to semitone scale.	Hypokinesia causes the reduced amplitude of vocal cord movements, leading to glottal incompetence.
**Speech timing**			
Slow SMR [syllable repetition]	DDKR	Diadochokinetic rate, defined as the number of syllable vocalizations per second.	Hypokinesia of speech apparatus makes the movements of articulators slower.
Prolonged pauses [monologue]	DPI	Duration of pause intervals, defined as the median length of pause intervals.	Hypokinesia of speech apparatus makes initiating of speech difficult, leading to prolonged pause intervals.

### Statistical analysis

An ad-hoc power analysis based on two-way analysis of variance with two covariates (GROUP and AGE) indicated a recommended minimum overall sample size of 52 for 4 groups (i.e., a minimum sample size of 13 per one group), given expected large effect size (Cohen’s *f* of 0.4) with the error probability *α* set at 0.05 and a false negative rate *β* set at 0.2 (i.e., power of 0.8)^59^. A two-way analysis of variance with GROUP (PD vs. controls) and AGE (younger vs. older) as between-subject factors was used to calculate differences for each speech dimension; GROUP per AGE interaction was used to assess possible phenotypic differences and to determine whether differences of speech performance in PD and controls are accentuated for a certain age group. For significant interactions, Bonferroni post-hoc tests were used to explore pairwise differences between four examined groups. To explore hypothesis of possible relation between speech and axial gait symptoms, Pearson’s correlation was used to test for associations between acoustic features and the bradykinesia, rigidity, or PIGD subscores. A two-tailed *p*-value < 0.05 was considered the threshold for statistically significant differences in all analyses.

### Reporting summary

Further information on research design is available in the Nature Research Reporting Summary linked to this article.

## Supplementary information

Supplementary Data 1

Reporting Summary

## Data Availability

Individual participant data that underlie the findings of this study are available upon reasonable request from the corresponding author. The speech data are not publicly available due to their contain of information that could compromise the privacy of study participants.

## Code availability

The analyses were performed using a publicly available Dysarthria Analyzer (Czech Technical University in Prague, available at http://dysan.cz/).
