# Supplementary material for: Distinct patterns of speech disorder in early-onset and late-onset de-novo Parkinson’s disease
Source: NPJ Parkinsons Dis. 2021 Nov 11;7:98. doi: 10.1038/s41531-021-00243-1 (PMC8585880; doi:10.1038/s41531-021-00243-1)
Supplement: Supplementary file 2 — Reporting Summary [file 41531_2021_243_MOESM2_ESM.pdf]

nature portfolio

Corresponding author(s): Jan Rusz

Last updated by author(s): Aug 20, 2021

Reporting Summary

Nature Portfolio wishes to improve the reproducibility of the work that we publish. This form provides structure for consistency and transparency in reporting. For further information on Nature Portfolio policies, see our Editorial Policies and the Editorial Policy Checklist.

Statistics

For all statistical analyses, confirm that the following items are present in the figure legend, table legend, main text, or Methods section.

n/a	Confirmed	
☐	☒	The exact sample size (n) for each experimental group/condition, given as a discrete number and unit of measurement
☐	☒	A statement on whether measurements were taken from distinct samples or whether the same sample was measured repeatedly
☐	☒	The statistical test(s) used AND whether they are one- or two-sided<br>*Only common tests should be described solely by name; describe more complex techniques in the Methods section.*
☐	☒	A description of all covariates tested
☐	☒	A description of any assumptions or corrections, such as tests of normality and adjustment for multiple comparisons
☐	☒	A full description of the statistical parameters including central tendency (e.g. means) or other basic estimates (e.g. regression coefficient) AND variation (e.g. standard deviation) or associated estimates of uncertainty (e.g. confidence intervals)
☐	☒	For null hypothesis testing, the test statistic (e.g. F, t, r) with confidence intervals, effect sizes, degrees of freedom and P value noted<br>*Give P values as exact values whenever suitable.*
☒	☐	For Bayesian analysis, information on the choice of priors and Markov chain Monte Carlo settings
☒	☐	For hierarchical and complex designs, identification of the appropriate level for tests and full reporting of outcomes
☐	☒	Estimates of effect sizes (e.g. Cohen's d, Pearson's r), indicating how they were calculated

Our web collection on statistics for biologists contains articles on many of the points above.

Software and code

Policy information about availability of computer code

Data collection	No software was used.
Data analysis	All analyses were performed in MATLAB® (MathWorks, Natick, MA).

For manuscripts utilizing custom algorithms or software that are central to the research but not yet described in published literature, software must be made available to editors and reviewers. We strongly encourage code deposition in a community repository (e.g. GitHub). See the Nature Portfolio guidelines for submitting code & software for further information.

Data

Policy information about availability of data

All manuscripts must include a data availability statement. This statement should provide the following information, where applicable:
- Accession codes, unique identifiers, or web links for publicly available datasets
- A description of any restrictions on data availability
- For clinical datasets or third party data, please ensure that the statement adheres to our policy

Individual participant data that underlie the findings of this study are available upon reasonable request from the corresponding author. The speech data are not publicly available due to their contain of information that could compromise the privacy of study participants.

Field-specific reporting

Please select the one below that is the best fit for your research. If you are not sure, read the appropriate sections before making your selection.

☐ Life sciences ☒ Behavioural & social sciences ☐ Ecological, evolutionary & environmental sciences

For a reference copy of the document with all sections, see nature.com/documents/nr-reporting-summary-flat.pdf

Behavioural & social sciences study design

All studies must disclose on these points even when the disclosure is negative.

Study description	Quantitative cross-sectional study.
Research sample	This study is based on a large sample of 48 patients considering that only early-onset (less than 51 years) and late-onset (more than 69 years) age of onset was considered in de-novo Parkinson's disease The early onset Parkinson's disease group consisted of 24 patients (15 men) with a mean age of 45.1 (SD 5.4, range 34–52) years and the late onset Parkinson's disease group included 24 patients (14 men) with a mean age of 75.4 (SD 3.1, range 71–81) years. The young healthy control group consisted of 24 participants (15 men) with a mean age of 45.2 (SD 5.6, range 35–52) years, whereas old healthy control group included 24 participants (14 men) with a mean age of 75.5 (SD 3.2, range 71–81) years.
Sampling strategy	An ad-hoc power analysis based on two-way analysis of variance with two covariates (GROUP and AGE) indicated a recommended minimum overall sample size of 52 for 4 groups (i.e., a minimum sample size of 13 per one group), given expected large effect size (Cohen's f of 0.4) with the error probability α set at 0.05 and a false negative rate β set at 0.2 (i.e., power of 0.8). We included sample size of 96 subjects for 4 groups.
Data collection	Speech recordings were performed in a quiet room with a low ambient noise level using a head-mounted condenser microphone (Beyerdynamic Opus 55, Heilbronn, Germany) placed approximately 5 cm from the subject's mouth. Speech signals were sampled at 48 kHz with 16-bit resolution. Each subject was recorded during a single session with a speech specialist. Researcher was blind to the study hypothesis during data collection.
Timing	From 2016 to 2021.
Data exclusions	No data were excluded from the analyses.
Non-participation	No participants fullfilling inclusion/exlusion criteria stated within manuscript dropped from the analyses.
Randomization	The early onset Parkinson's disease group included patients with age at onset ≤ 50 years while late onset Parkinson's disease group consisted of patients with age at onset ≥ 70 years. In addition, young healthy control group age- and sex-matched to the early onset Parkinson's disease group and old healthy control group age- and sex-matched to the late onset Parkinson's disease group were enrolled.

Reporting for specific materials, systems and methods

We require information from authors about some types of materials, experimental systems and methods used in many studies. Here, indicate whether each material, system or method listed is relevant to your study. If you are not sure if a list item applies to your research, read the appropriate section before selecting a response.

Materials & experimental systems

n/a	Involved in the study	
☒	☐	Antibodies
☒	☐	Eukaryotic cell lines
☒	☐	Palaeontology and archaeology
☒	☐	Animals and other organisms
☐	☒	Human research participants
☒	☐	Clinical data
☒	☐	Dual use research of concern

Methods

n/a	Involved in the study	
☒	☐	ChIP-seq
☒	☐	Flow cytometry
☒	☐	MRI-based neuroimaging

Human research participants

Policy information about studies involving human research participants

Population characteristics	See above.

Recruitment	The Parkinson's disease patients were recruited at General University Hospital in Prague. The control subjects were recruited from the general community through advertisements. No selection bias was present.
Ethics oversight	The study was approved by the Ethics Committee of the General University Hospital in Prague, Czech Republic and have therefore been performed in accordance with the ethical standards laid down in the 1964 Declaration of Helsinki and its later amendments. All participants provided written, informed consent to the neurological examination and recording procedure.

Note that full information on the approval of the study protocol must also be provided in the manuscript.
